# Retrospective analysis of online news on femicide in Türkiye (2018–2022)

**DOI:** 10.1590/1980-220X-REEUSP-2025-0080en

**Published:** 2025-11-17

**Authors:** Sema Çifçi, Beril Nisa Yașar

**Affiliations:** 1 Mardin Artuklu University, Faculty of Health Sciences, Nursing Department, Mardin, Türkiye.

**Keywords:** Homicide, Periodical, Violence Against Women, News, Nursing, Homicídio, Publicação Periódica, Violência contra a Mulher, Notícias, Enfermagem

## Abstract

**Objective:**

The aim of this study is to examine the femicides covered in online newspapers in Türkiye between 2018 and 2022, to identify the characteristics of perpetrators and victims, and to reveal the reasons for these murders and how they took place.

**Method:**

News published in 19 online newspapers was analyzed by using the Google Search Engine in a retrospective-descriptive design. The data were evaluated based on variables such as the age of the victims, marital status, family type, place of murder, cause and manner of murder, and the relationship of the perpetrators with the victims. Data were analyzed using SPSS 24.

**Results:**

We found that most femicides occurred in 2020, were committed by husbands (34.2%) using firearms (43.3%), and were driven by arguments (45.5%). Victims were predominantly 20–40 years old, married, and housewives.

**Conclusion:**

In order to prevent violence against women, it is important to raise social awareness, strengthen legal regulations and develop protective policies. Although the findings provide insight into the media visibility and demographic trends of femicides in Türkiye, they need to be supported by more comprehensive epidemiological studies.

## RESUMO

**Objetivo:**

O objetivo deste estudo é examinar os feminicídios cobertos por jornais online na Turquia entre 2018 e 2022, identificar as características dos perpetradores e das vítimas e revelar as razões por trás desses assassinatos e como eles ocorreram.

**Método:**

As notícias publicadas em 19 jornais online foram analisadas utilizando o motor de busca Google, num desenho retrospectivo-descritivo. Os dados foram avaliados com base em variáveis como a idade das vítimas, estado civil, tipo de família, local do homicídio, causa e forma do homicídio e a relação dos perpetradores com as vítimas. Os dados foram analisados utilizando o SPSS 24.

**Resultados:**

Constatou-se que a maioria dos feminicídios ocorreu em 2020, foi cometida por maridos (34,2%) com armas de fogo (43,3%) e motivada por discussões (45,5%). As vítimas tinham predominantemente entre 20 e 40 anos, eram casadas e donas de casa.

**Conclusão:**

Para prevenir a violência contra as mulheres, é importante aumentar a consciência social, fortalecer as regulamentações legais e desenvolver políticas de proteção. Embora as descobertas forneçam informações sobre a visibilidade da mídia e as tendências demográficas dos feminicídios na Turquia, elas precisam ser apoiadas por estudos epidemiológicos mais abrangentes.

## INTRODUCTION

Violence against women is considered one of the most serious problems for women and girls on a global scale. Based on the definition of violence against women as “Any act of violence that causes or is likely to cause physical, sexual or psychological harm or suffering to women”^(1, p. 2)^, it is understood as a widespread problem that creates individual and social impacts. Although violence against women has very serious impacts on all segments of society, women who are deprived of their right to life pay the heaviest price in this context^(2)^, which causes physical, sexual, or psychological harm to women, and creates serious effects on both the individual and the social level.

Studies that were conducted previously by the World Health Organization (WHO) reported that rates of physical or sexual violence vary between 15% and 71% by country^(3)^. The prevalence of physical and/or sexual violence within the family was found to be 42% in Türkiye. This demonstrates that violence must be addressed as a social concern^(4)^. As an international solidarity organization, the Independent Communication Network reported that 286 women and girls were killed in Türkiye in 2017, 418 women were victims of violence, at least 255 women and 17 girls, including baby girls, were killed and 380 women were injured in 2018^(5)^. Based on the data of the “We Will Stop Femicide Platform”, 383 women were killed in the first ten months of 2019^(6)^.

The media plays significant roles in ensuring social change and transformation by rapidly delivering information and news to the masses, presenting it as visual, auditory, and written text, and having a structure that can appeal to and interest every target audience. In this respect, the media increases the effects of violent acts and might even cause individuals to internalize violence^(7)^. The way violence is reflected is as important as the causes of violence in combating it^(8)^.

Femicide is a problem on a global scale and deeply affects not only women’s lives but also the social structure and the struggle for gender equality. Correctly analyzing news about femicide in the media is critical to understanding how these murders are perceived in society and how the media shapes this perception. Depending on how the media presents the causes, perpetrators, and victims of femicide, it can either increase public awareness or reinforce stereotypes that normalize violence. Studies conducted previously on media representations of femicide shed light on the development of effective policies and strategies to combat violence.

Most of the studies on femicides in Türkiye are based on judicial records, police data and official statistics. While these studies provide numerical findings, they do not sufficiently focus on the social representation and narrative forms of the events through media reports. The limited number of comprehensive analyses on how femicides are portrayed in the media points to an important gap in the field. The aim of this study is to examine the femicides covered in online newspapers in Türkiye between 2018 and 2022, to identify the characteristics of perpetrators and victims, and to reveal the reasons for these murders and how they took place.

## MATERIAL AND METHODS

### Design of Study

The study had a descriptive cross-sectional retrospective design.

### Local

The study did not distinguish between regions or cities and covered the online news reported within the borders of Türkiye and reflected in the media.

### Population

This research includes all online news reports of femicide occurring within Türkiye, without limiting to specific regions or cities. According to the Turkish Press Association, there are over 226 active online newspapers in Türkiye as of 2022^(9)^.

### Selection Criteria

- News of femicide in Sabah, Milliyet, Hürriyet, Cumhuriyet, Sözcü, CNN Türk, Haber Türk, Evrensel Net, Bölge Gündem, Sol Gazete, Yeni Yaşam Gazetesi, Gazete Duvarı, Birgün Gazetesi, Posta, Star, Mynet news sites.
- The news published between 01.01.2018 and 31.12.2022.
- The incident in the news taking place in Türkiye.

### Exclusion Criteria

Femicide cases reported in the print media between 01.01.2018 and 31.12.2022 were not included in the study.

### Sample Definition

The data were obtained with the search conducted with “femicide” and related keywords. The news examined was grouped based on variables such as the causes of the murders, the profiles of the perpetrators, the places where the events took place, and the temporal distribution. The news items used in this study were compiled from 19 online newspapers with widespread access in Türkiye. The selection of newspapers was based on criteria such as having high circulation on a national scale, having online archives that are accessible and suitable for retrospective scanning, providing continuity in news production and representing different ideological/political lines. In this context, newspapers belonging to center-right, center-left and independent media organizations were included.

### Data Collection

The news items about femicide in online newspapers in Türkiye between 2018 and 2022 were examined in the study. The news examined was grouped based on variables such as the causes of the murders, the profiles of the perpetrators, the places where the events took place, and the temporal distribution. In this study, a structured data collection form developed by the research team was used to systematically extract the identified variables from the news on femicide. The data collection process was conducted by two independent researchers with academic experience in the field and the information obtained was double-checked to increase the reliability of the data. The researchers have expertise in gender-based violence and media analysis. The entire process was planned and reported in line with the STROBE checklist, ensuring that the study complied with scientific reporting standards for observational research.

### Data Analysis

The SPSS 24 software was used for data analysis, and the data were evaluated with numbers and percentage distributions.

### Ethical Aspects

Before starting the study, ethics committee approval (2024/2–3, 13.03.2024) was obtained from Mardin Artuklu University Non-Interventional Ethics Committee. We have anonymized the news, paying attention to privacy and personal data and deciphering names.

## RESULTS

The findings were obtained for a total of 1,982 femicide news items published in online newspapers in Türkiye. The tables include demographic information of the victims and perpetrators, the reasons for the murders, the way they occurred, and their spatial and temporal distribution.

It was found in the present study that a total of 1982 women were murdered in the last 5 years (Table 1).

**Table 1:** Femicide by years – Türkiye, 2024.

Years	n		%
2018	361		18.2
2019	409		20.6
2020	421		21.2
2021	404		20.4
2022	387		19.5
**Total**	1982		100.0

The age ranges of the murdered women were found to be 20–40 years old (52.6%), 40–60 years old (22.2%), 0–20 years old (13.9%), 60–80 years old (11.3%), 60.3% were married and 39.7% were single, 60.3% were in nuclear families, 39.7% were in extended families, and 68.7% were housewives. When their educational status was examined, 77.9% were primary school graduates, 20.8% were secondary school graduates, 1% were high school graduates, and 0.4% had a bachelor’s degree (Table 2).

**Table 2:** Demographic Data of Murdered Women – Türkiye, 2024.

Demographic Data	n	%
Age Range		
0–20	276	13.9
20–40	1043	52.6
40–60	440	22.2
60–80	223	11.3
Marital Status		
Married	1142	60.3
Single	840	39.7
Family Type		
Extended family	786	60.3
Nuclear family	1196	39.7
Job		
Housewife	1361	68.7
Officer	599	30.2
Other	22	1.1
Educational Status		
Primary school	1543	77.9
Middle school	412	20.8
High school	19	1.0
Undergraduate	8	0.4
**Total**	1982	100.0

A total of 34.2% of women were killed by their husbands, 20.7% by non-identified persons, 10.6% by their sons, 9.6% by their lovers, 6.2% by their ex-husbands, 5% by someone they knew, 4.6% by their relatives, 3.9% by their fathers, 3.2% by someone they did not know, 1.5% by their grooms, 0.3% by their neighbors, 0.2% by their fiancés and 64.7% of the men who used violence were employed and 33.2% were unemployed (Table 3).

**Table 3:** Characteristics of Persons Who Committed Femicides – Türkiye, 2024.

The Person Who Committed the Murder	n	%
Husband	678	34.2
Son	210	10.6
Someone She Does Not Know	64	3.2
Her father	77	3.9
Her lover	190	9.6
Her relative	92	4.6
Fiancé	3	0.2
Her neighbor	6	0.3
Her groom	30	1.5
Someone He Knows	99	5.0
Ex-husband	123	6.2
Non-defined	410	20.7
Professions of the Men Who Committed the Murder		
Unemployed	659	33.2
Worker	1282	64.7
Other	41	2.1
**Total**	1982	100.0

Also, 43.3% of women were killed by firearms, 19.7% by cutting tools, 6.2% by strangulation, 4.8% by beating, 2.6% by falling from a height, 1.8% by burning, 1.6% by hanging, 0.1% by drugging, 19.9% by a non-defined method or methods, 45.5% were killed because of an argument, 13.4% because of rejection, 12.4% because of a divorce request, 11.5% because of jealousy, 5% because they were trying to protect someone (their sister, brother, mother), 4.1% because of hostility, 2.9% were killed accidentally, 2.8% because of inheritance, and 2.4% because of money. The regions where the murders were committed were the Marmara Region (28.7%), the Mediterranean Region (17.2%), the Central Anatolia Region (14.7%), the Aegean Region (10.6%), the Black Sea Region (9.7%), the Southeastern Anatolia Region (9.6%) and the Eastern Anatolia Region (9.5%), respectively. It was also found that most of the femicides (61.7%) occurred during the day (Table 4).

**Table 4:** Information on Femicides – Turkiye, 2024.

How the Murder Was Committed	n	%
Cutting tool	390	19.7
Firearm	858	43.3
By hanging	32	1.6
By drowning	123	6.2
By being beaten	96	4.8
Burning	35	1.8
Falling from a height	52	2.6
By giving drugs	2	0.1
Non-defined	394	19.9
Reasons for Murder		
Jealousy	227	11.5
Argument	901	45.5
Animosity	82	4.1
Trying to protect someone	100	5.0
Rejection	266	13.4
Request for divorce	246	12.4
Because of money	48	2.4
By accident	57	2.9
Heritage	55	2.8
Area Where Murder Was Committed		
Eastern Anatolia	189	9.5
Southeastern Anatolia	191	9.6
Mediterranean	340	17.2
Black Sea	192	9.7
Marmara	569	28.7
Aegean	211	10.6
Central Anatolia	290	14.7
Time of the Murder		
Daytime	1223	61.7
Night	759	38.3
**Total**	1982	100.0

## DISCUSSION

In the present study, the news items about murders against women in the online versions of Sabah, Milliyet, Hürriyet, Cumhuriyet, Sözcü, CNN Türk, Haber Türk, Evrensel.Net, Bölge Gündem, Sol Gazete, Yeni Yaşam Gazetesi, Gazete Duvar, Birgün Gazetesi, Posta, Star and Mynet News websites were examined retrospectively.

Like all forms of gender-based violence against women, femicide is a concern affecting every country in the world. The United Nations Office on Drugs and Crime’s Global Homicide Report reported that femicides occur most frequently in Asian and African countries and least frequently in European countries^(10)^. According to 2019 data from the Organization for Economic Co-operation and Development, Türkiye has the highest rate of violence against women (38%) although Canada is the country with the lowest rate of violence against women (1.9%)^(11)^. The “We Will Stop Femicide Platform” reported that there were 112 femicides and 79 suspicious deaths of women as of June 2021, and 300 femicides and 171 suspicious deaths of women in 2020^(6)^. Based on the results of the 2014 Domestic Violence Against Women Survey, the rate of women in Türkiye who were victims of physical violence by their husbands or partners at some point in their lives was 36.0%^(12)^. In a study conducted in Türkiye, it was reported that news on violence increased exponentially from 2013 to 2016^(13)^.

In the present study, it was found that a total of 1982 women were murdered in the newspapers examined in the last 5 years. When calculated by years, 361 women were killed in 2018, 409 in 2019, 421 in 2020, 404 in 2021, and 387 in 2022 (Table 1). Based on the data obtained in the study, it can be argued that femicides were committed at a rate similar to the literature between 2018 and 2022. In 2020, approximately 47.000 women and girls were killed by their intimate partners or other family members in the world. In other words, on average, a woman or girl is killed by a member of her own family every 11 minutes^(14)^. Estimates of lifetime prevalence of intimate partner violence are 20% in the Western Pacific, 22% in high-income countries and Europe, 25% in the WHO Regions of the Americas, 33% in the WHO Africa region, 31% in the WHO Eastern Mediterranean region, and 33% in the WHO Southeast Asia region. More than a quarter of women aged 15–49 years worldwide have experienced physical and/or sexual violence by an intimate partner at least once in their lifetime^(15)^.

Consistent with the literature data, the women killed were mostly between the ages of 20–40 (52.6%) (Table 2). In studies conducted in Türkiye at different times, the age ranges of femicides were determined as 21–50^(16)^, 25–39^(13,17)^, 10–20^(18)^, 18–40^(19)^ and 18–30^(20)^ respectively. According to studies, it can be said that women are exposed to violence in their youth and adulthood.

Previous studies report that women who are most exposed to violence are married^(20)^ or unmarried women^(11,17, 18, 19)^. In the present study, 60.3% of the women who were killed were married and 39.7% were single (Table 2). It can be argued that women were victims of femicide to violence or killed regardless of their marital status.

A total of 60.3% of the murdered women were from nuclear families and 39.7% were from extended families in the present study (Table 2). Factors such as the fact that girls and boys were raised according to patriarchal values, incompatibility of spouses, an environment prone to violence, and low socioeconomic status may have affected the increase in violence against women.

Previous studies also show that women victims of femicide to violence were employed in a job that generated income^(19)^, their occupation was unknown^(21)^ or their occupation was not reported^(20)^. In the present study, it was determined that 68.7% of the women who were killed were housewives (Table 2).

Different studies provide important findings regarding the educational status of women victims of violence. In one study, it was found that the educational status of 87% of women victims of violence was not included^(19)^, another study revealed that 77.8% of illiterate women and 51.1% of primary school graduates were victims of violence^(22)^. In another study, it was reported that only 10.5% of women victims of violence were university graduates^(23)^. Studies also indicate that the educational status of women victims of femicide to violence is largely not reported in the news in newspapers^(20)^. In the present study, it was found that a significant portion of the women victims of femicide to the news (77.9%) were primary school graduates (Table 2).

Also, 34.2% of the women were killed by their husbands, 20.7% by non-defined persons, 10.6% by their sons, 9.6% by their lovers, and 6.2% by their ex-husbands in the present study (Table 3). Studies also report that the person who commits femicide is most often the woman’s partner (husband, ex-wife, fiancé, lover)^(2,17,24, 25, 26)^. Accordingly, it can be concluded that women are most victims of femicide to violence by people with whom they have established emotional closeness.

In the present study, the highest rate of women was killed by firearms, 19.7% by cutting tools, and 6.2% by strangulation, respectively (Table 4). Studies on the incidents of violence reported in newspapers on different dates show that physical force is mostly used as a means of violence against women, followed by firearms^(17,19,20)^. In another study based on newspaper reports during the pandemic period, it was found that women were mostly killed with firearms^(2)^. Based on these results, it can be said that women were killed by firearms, force, brute force, and injury.

In previous studies, women were killed for reasons such as the woman’s desire for divorce or separation^(25,26)^, jealousy, infidelity, honor and separation, arguments^(2)^, suspicion of cheating on her husband, requesting divorce or separation^(24)^, etc.^(13,17,20)^. In the present study, 45.5% of the women were killed because of arguments, 13.4% because of rejection, 12.4% because of requesting divorce, and 11.5% because of jealousy (Table 4). The traditional understanding accepted in society as the justification for femicide is also responsible for the suicides and murders of many women^(2,27)^.

When the rates of exposure to violence in Türkiye were examined by regions, it was determined that physical violence is most common in the Central Anatolia Region (43%), sexual violence was most common in the Northeastern Anatolia Region (16%), and emotional violence was most common in the Western Anatolia Region (54%)^(28)^. In the present study, the regions where the murders were committed were determined as Marmara Region (28.7%), Mediterranean Region (17.2%), Central Anatolia Region (14.7%), Aegean Region (10.6%), Black Sea Region (9.7%), Southeastern Anatolia Region (9.6%) and Eastern Anatolia Region (9.5%), respectively (Table 4). This suggests that the Marmara Region may be related to being a migrant-receiving, cosmopolitan, and having a high population density. However, it is recommended to further investigate why certain regions have higher rates, taking into account factors such as urbanisation, socioeconomic status and cultural context.

In a study conducted in 2021, it was found that women were more exposed to violence during the day^(2)^, while another study revealed that the highest rate of violence was experienced at night^(20)^. In the present study, it was found that the highest rate of femicides (61.7%) occurred during the day (Table 4).

It is necessary to emphasize that violence against women in Türkiye is carried out by males. Previous studies show that 29% of those who perpetrate violence have a profession and 71% do not have a job that brings income^(21)^. In a study conducted in 2005, it was reported that 37.8% of the men who perpetrated violence did not have a permanent job^(29)^. In our study, 33.2% of the men who perpetrated violence were found to be unemployed (Table 3).

The data of the study reveal that femicide is an important public health problem in Türkiye and urgent measures need to be taken. Prevention of violence against women is vital not only for the protection of individual rights, but also for the protection of the general health and welfare of the society.

Healthcare professionals, especially nurses, are important actors in terms of early intervention, guidance and awareness- raising, as they have direct contact with women victims of violence. Therefore, training programs for health personnel should be increased. In addition, news on femicide in the media should be presented in a manner sensitive to human dignity and women’s rights and should be supported by legal regulations.

### Limitations

The data of the study were obtained only from newspapers in Türkiye that were freely accessible online between 2018–2022. For this reason, news about femicide in paid or limited access, printed media, or different news sources could not be examined. The lack of or insufficient information about some events in the evaluated news limited the detailed analysis. The statistics obtained are limited to the information published in newspapers and may not fully overlap with the real data.

This study is based solely on reports of femicide in online news websites. Therefore, they may not reflect the completeness, accuracy and coverage provided by official epidemiological data. These results cannot be generalised to the society. The data sources of other studies used in comparative evaluations may have been obtained from more systematic sources such as official records or forensic medicine reports. These methodological differences may limit direct comparison of the results of our study. It is necessary to expand the limitations on data sources and to recognise that online media may differ from print media in terms of reporting and audience.

The data collection tool used in this study was not pre-tested and validity analysis was not performed. This may be a limitation in terms of the reliability of the data obtained. However, the variables were clearly defined and the data were collected by two independent researchers to reduce the margin of error.

## CONCLUSION

A total of 1,982 news items on femicide were identified in the study. In the present study that examined femicides in newspaper reports, it was found that women were killed the most in 2020 by their husbands, with firearms, because of arguments, as primary school graduates, married and housewives, in extended families, in the Marmara region, between the ages of 20–40, during the daytime, and by working men. The findings reveal that femicides are based on multidimensional causes and that comprehensive prevention strategies need to be developed.

## Data Availability

The full dataset supporting the findings of this study is available upon request to the corresponding author.
