# Supplementary material for: Retrospective analysis of online news on femicide in Türkiye (2018–2022)
Source: Rev Esc Enferm USP. 2025 Nov 17;59:e20250080. doi: 10.1590/1980-220X-REEUSP-2025-0080en (PMC12625991; doi:10.1590/1980-220X-REEUSP-2025-0080en)
Supplement: Translation

# Translation: Análisis retrospectivo de las noticias en línea sobre feminicidios en Turquía (2018–2022)

**DOI:** 10.1590/1980-220X-REEUSP-2025-0080es

**Authors:** Sema Çifçi, Beril Nisa Yașar

**Affiliations:** 1 Mardin Artuklu University, Faculty of Health Sciences, Nursing Department, Mardin, Türkiye.

**Keywords:** Homicidio, Publicación Periódica, Violencia contra la Mujer, Noticias, Enfermería

## Abstract

**Objetivo:**

El objetivo de este estudio es examinar los feminicidios cubiertos en los periódicos en línea de Turquía entre 2018 y 2022, identificar las características de los autores y las víctimas, y revelar las razones de estos asesinatos y cómo se produjeron.

**Método:**

Se analizaron las noticias publicadas en 19 periódicos en línea utilizando el motor de búsqueda Google, con un diseño retrospectivo-descriptivo. Los datos se evaluaron en función de variables como la edad de las víctimas, el estado civil, el tipo de familia, el lugar del asesinato, la causa y la forma del asesinato, y la relación de los autores con las víctimas. Los datos se analizaron utilizando el programa SPSS 24.

**Resultados:**

Se observó que la mayoría de los feminicidios se produjeron en 2020, fueron cometidos por maridos (34,2%) con armas de fuego (43,3%) y motivados por discusiones (45,5%). Las víctimas eran en su mayoría mujeres de entre 20 y 40 años, casadas y amas de casa.

**Conclusión:**

Para prevenir la violencia contra las mujeres, es importante sensibilizar a la sociedad, reforzar la normativa legal y desarrollar políticas de protección. Aunque los resultados proporcionan información sobre la visibilidad mediática y las tendencias demográficas de los feminicidios en Turquía, deben respaldarse con estudios epidemiológicos más exhaustivos.

## INTRODUCCIÓN

La violencia contra las mujeres se considera uno de los problemas más graves para las mujeres y las niñas a escala mundial. Según la definición de violencia contra las mujeres como “cualquier acto de violencia que cause o pueda causar daño o sufrimiento físico, sexual o psicológico a las mujeres”^(1, p. 2)^, se entiende como un problema generalizado que tiene repercusiones individuales y sociales. Aunque la violencia contra las mujeres tiene repercusiones muy graves en todos los segmentos de la sociedad, las mujeres que se ven privadas de su derecho a la vida son las que pagan el precio más alto en este contexto^(2)^, lo que causa daños físicos, sexuales o psicológicos a las mujeres y tiene graves efectos tanto a nivel individual como social.

Estudios realizados anteriormente por la Organización Mundial de la Salud (OMS) revelaron que las tasas de violencia física o sexual varían entre el 15% y el 71% según el país^(3)^. Se constató que la prevalencia de la violencia física y/o sexual dentro de la familia era del 42% en Turquía. Esto demuestra que la violencia debe abordarse como una preocupación social^(4)^. Como organización de solidaridad internacional, la Red de Comunicación Independiente informó de que 286 mujeres y niñas fueron asesinadas en Turquía en 2017, 418 mujeres fueron víctimas de violencia, al menos 255 mujeres y 17 niñas, incluidas bebés, fueron asesinadas y 380 mujeres resultaron heridas en 2018^(5)^. Según los datos de la plataforma “We Will Stop Femicide”, 383 mujeres fueron asesinadas en los primeros diez meses de 2019^(6)^.

Los medios de comunicación desempeñan un papel importante a la hora de garantizar el cambio y la transformación social, ya que transmiten rápidamente información y noticias a las masas, las presentan en formato visual, auditivo y escrito, y cuentan con una estructura capaz de atraer e interesar a todos los públicos objetivo. En este sentido, los medios de comunicación aumentan los efectos de los actos violentos e incluso pueden llevar a las personas a interiorizar la violencia^(7)^. La forma en que se refleja la violencia es tan importante como sus causas a la hora de combatirla^(8)^.

El feminicidio es un problema a escala mundial y afecta profundamente no solamente la vida de las mujeres, sino también a la estructura social y a la lucha por la igualdad de género. Analizar correctamente las noticias sobre feminicidios en los medios de comunicación es fundamental para comprender cómo se perciben estos asesinatos en la sociedad y cómo los medios de comunicación configuran esta percepción. Dependiendo de cómo los medios de comunicación presenten las causas, los autores y las víctimas de los feminicidios, pueden aumentar la conciencia pública o reforzar los estereotipos que normalizan la violencia.

Los estudios realizados anteriormente sobre la representación del feminicidio en los medios de comunicación arrojan luz sobre el desarrollo de políticas y estrategias eficaces para combatir la violencia. La mayoría de los estudios sobre feminicidios en Turquía se basan en registros judiciales, datos policiales y estadísticas oficiales. Si bien estos estudios proporcionan resultados numéricos, no se centran suficientemente en la representación social y las formas narrativas de los acontecimientos a través de los reportajes de los medios de comunicación. El número limitado de análisis exhaustivos sobre cómo se retratan los feminicidios en los medios de comunicación pone de manifiesto una importante laguna en este ámbito.

El objetivo de este estudio es examinar los feminicidios cubiertos en los periódicos en línea de Turquía entre 2018 y 2022, identificar las características de los autores y las víctimas, y revelar las razones de estos asesinatos y cómo se produjeron.

## MATERIAL Y MÉTODOS

### Diseño del Estudio

El estudio tuvo un diseño descriptivo transversal retrospectivo.

### Local del Estudio

El estudio no distinguió entre regiones o ciudades y abarcó las noticias en línea publicadas dentro de las fronteras de Turquía y reflejadas en los medios de comunicación.

### Población

Esta investigación incluye todas las noticias en línea sobre feminicidios ocurridos en Turquía, sin limitarse a regiones o ciudades específicas. Según la Asociación de Prensa Turca, en 2022 hay más de 226 periódicos en línea activos en Turquía^(9)^.

### Criterios de Selección

- Noticias sobre feminicidios en los sitios web de noticias Sabah, Milliyet, Hürriyet, Cumhuriyet, Sözcü, CNN Türk, Haber Türk, Evrensel Net, Bölge Gündem, Sol Gazete, Yeni Yaşam Gazetesi, Gazete Duvarı, Birgün Gazetesi, Posta, Star y Mynet.
- Noticias publicadas entre el 1 de enero de 2018 y el 31 de diciembre de 2022.
- Incidentes recogidos en las noticias que tuvieron lugar en Turquía.

### Criterios de Exclusión

No se incluyeron en el estudio los casos de feminicidio publicados en la prensa escrita entre el 1 de enero de 2018 y el 31 de diciembre de 2022.

### Definición de la Muestra

Los datos se obtuvieron mediante una búsqueda con las palabras clave “feminicidio” y otras relacionadas. Las noticias examinadas se agruparon en función de variables como las causas de los asesinatos, los perfiles de los autores, los lugares donde tuvieron lugar los hechos y la distribución temporal. Las noticias utilizadas en este estudio se recopilaron de 19 periódicos en línea con amplia difusión en Turquía. La selección de los periódicos se basó en criterios como tener una alta circulación a escala nacional, disponer de archivos en línea accesibles y adecuados para su consulta retrospectiva, ofrecer continuidad en la producción de noticias y representar diferentes líneas ideológicas/políticas. En este contexto, se incluyeron periódicos pertenecientes a organizaciones mediáticas de centro-derecha, centro-izquierda e independientes.

### Recopilación de Datos

En el estudio se examinaron las noticias sobre feminicidios publicadas en periódicos en línea en Turquía entre 2018 y 2022. Las noticias examinadas se agruparon en función de variables como las causas de los asesinatos, los perfiles de los autores, los lugares donde tuvieron lugar los hechos y la distribución temporal. En este estudio se utilizó un formulario estructurado de recopilación de datos elaborado por el equipo de investigación para extraer sistemáticamente las variables identificadas de las noticias sobre feminicidios. El proceso de recolección de datos fue llevado a cabo por dos investigadores independientes con experiencia académica en el campo y la información obtenida se verificó dos veces para aumentar la fiabilidad de los datos. Los investigadores tienen experiencia en violencia de género y análisis de medios de comunicación. Todo el proceso se planificó y documentó de acuerdo con la lista de verificación STROBE, lo que garantizó que el estudio cumpliera con las normas de información científica para la investigación observacional.

### Análisis de Datos

Se utilizó el software SPSS 24 para el análisis de los datos, que se evaluaron con números y distribuciones porcentuales.

### Aspectos Éticos

Antes de iniciar el estudio, se obtuvo la aprobación del comité de ética (2024/2-3, 13.03.2024) del Comité de Ética No Intervencionista de la Universidad Artuklu de Mardin. Hemos anonimizado las noticias, prestando atención a la privacidad y los datos personales y descifrando los nombres.

## RESULTADOS

Los resultados se obtuvieron a partir de un total de 1982 noticias sobre feminicidios publicadas en periódicos digitales de Turquía. Las tablas incluyen información demográfica sobre las víctimas y los autores, los motivos de los asesinatos, la forma en que se produjeron y su distribución espacial y temporal.

En el presente estudio se constató que un total de 1982 mujeres fueron asesinadas en los últimos 5 años (Tabla 1).

**Tabla 1:** Feminicidios por años – Turquía, 2024.

Años	n		%
2018	361		18,2
2019	409		20,6
2020	421		21,2
2021	404		20,4
2022	387		19,5
**Total**	1982		100,0

Se encoontró que las edades de las mujeres asesinadas oscilaban entre los 20 y los 40 años (52,6%), los 40 y los 60 años (22,2%), los 0 y los 20 años (13,9%) entre 60 y 80 años (11,3%), el 60,3% estaban casadas y el 39,7% eran solteras, el 60,3% vivían en familias nucleares, el 39,7% en familias extensas y el 68,7% eran amas de casa. Al examinar su nivel educativo, el 77,9% eran graduados de primaria, el 20,8% eran graduados de secundaria, el 1% eran graduados de bachillerato y el 0,4% tenían una licenciatura (Tabla 2).

**Tabla 2:** Datos demográficos de las mujeres asesinadas – Turquía, 2024.

Datos demográficos	n	%
Rango de edad		
0–20	276	13,9
20–40	1043	52,6
40–60	440	22,2
60–80	223	11,3
Estado civil		
Casada	1142	60,3
Soltera	840	39,7
Tipo de familia		
Familia extensa	786	60,3
Familia nuclear	1196	39,7
Empleo		
Ama de casa	1361	68,7
Oficial	599	30,2
Otros	22	1,1
Nivel educativo		
Escuela primaria	1543	77,9
Secundaria 1er. ciclo	412	20,8
Secundaria 2o. ciclo	19	1,0
Licenciatura	8	0,4
**Total**	1982	100,0

Un total del 34,2% de las mujeres fueron asesinadas por sus maridos, el 20,7% por personas no identificadas, el 10,6% por sus hijos, el 9,6% por sus amantes, el 6,2% por sus exmaridos, el 5% por alguien que conocían, el 4,6% por sus familiares, el 3,9% por sus padres, el 3,2% por alguien que no conocían, el 1,5% por sus novios e es, el 0,3% por sus vecinos, el 0,2% por sus prometidos y el 64,7% de los hombres que ejercieron violencia tenían empleo y el 33,2% estaban desempleados (Tabla 3).

**Tabla 3:** Características de las personas que cometieron feminicidios – Turquía, 2024.

La persona que cometió el asesinato	n	%
Marido	678	34,2
Hijo	210	10,6
Alguien que no conoce	64	3,2
Su padre	77	3,9
Su amante	190	9,6
Su pariente	92	4,6
Prometido	3	0,2
Su vecino	6	0,3
Su novio	30	1,5
Alguien que conoce	99	5,0
Exmarido	123	6,2
Sin definir	410	20,7
Profesiones de los hombres que cometieron el asesinato		
Desempleado	659	33,2
Trabajador	1282	64,7
Otros	41	2,1
**Total**	1982	100,0

Además, el 43,3% de las mujeres fueron asesinadas con armas de fuego, el 19,7% con herramientas cortantes, el 6,2% por estrangulamiento, el 4,8% por golpes, el 2,6% por caídas desde altura, el 1,8% por quemaduras, el 1,6% por ahorcamiento, el 0,1% por envenenamiento, el 19,9% por un método o métodos no definidos, el 45,5% fueron asesinadas a causa de una discusión, el 13,4% a causa de un rechazo, el 12,4% a causa de una solicitud de divorcio, el 11,5% a causa de los celos, el 5% porque intentaban proteger a alguien (su hermana, hermano, madre), el 4,1% por hostilidad, el 2,9% fueron asesinadas accidentalmente, el 2,8% por una herencia y el 2,4% por dinero. Las regiones donde se cometieron los asesinatos fueron la región de Mármara (28,7%), la región mediterránea (17,2%), la región de Anatolia Central (14,7%), la región del Egeo (10,6%), la región del Mar Negro (9,7%), la región del sudeste de Anatolia (9,6%) y la región del este de Anatolia (9,5%), respectivamente. También se descubrió que la mayoría de los feminicidios (61,7%) se produjeron durante el día (Tabla 4).

**Tabla 4:** Información sobre feminicidios – Turquía, 2024.

Cómo se cometió el asesinato	n	%
Herramienta cortante	390	19,7
Arma de fuego	858	43,3
Por ahorcamiento	32	1,6
Por ahogamiento	123	6,2
Por golpes	96	4,8
Quemado	35	1,8
Caída desde una altura	52	2,6
Administrando drogas	2	0,1
No definido	394	19,9
Motivos del asesinato		
Celos	227	11,5
Discusión	901	45,5
Animosidad	82	4,1
Intentar proteger a alguien	100	5,0
Rechazo	266	13,4
Solicitud de divorcio	246	12,4
Por motivos económicos	48	2,4
Por accidente	57	2,9
Patrimonio	55	2,8
Zona donde se cometió el asesinato		
Anatolia Oriental	189	9,5
Anatolia sudoriental	191	9,6
Mediterráneo	340	17,2
Mar Negro	192	9,7
Mármara	569	28,7
Egeo	211	10,6
Anatolia Central	290	14,7
Hora del asesinato		
Durante el día	1223	61,7
Noche	759	38,3
**Total**	1982	100,0

## DISCUSIÓN

En el presente estudio, se examinaron retrospectivamente las noticias sobre asesinatos de mujeres publicadas en las versiones en línea de los sitios web Sabah, Milliyet, Hürriyet, Cumhuriyet, Sözcü, CNN Türk, Haber Türk, Evrensel.Net, Bölge Gündem, Sol Gazete, Yeni Yaşam Gazetesi, Gazete Duvar, Birgün Gazetesi, Posta, Star y Mynet News.

Al igual que todas las formas de violencia de género contra las mujeres, el feminicidio es un problema que afecta a todos los países del mundo. El Informe mundial sobre homicidios de la Oficina de las Naciones Unidas contra la Droga y el Delito señaló que los feminicidios se producen con mayor frecuencia en los países asiáticos y africanos y con menor frecuencia en los países europeos^(10)^. Según datos de 2019 de la Organización para la Cooperación y el Desarrollo Económicos, Turquía tiene la tasa más alta de violencia contra las mujeres (38%), mientras que Canadá es el país con la tasa más baja (1,9%)^(11)^. La plataforma “We Will Stop Femicide” informó de que, hasta junio de 2021, se habían producido 112 feminicidios y 79 muertes sospechosas de mujeres, y 300 feminicidios y 171 muertes sospechosas de mujeres en 2020^(6)^. Según los resultados de la Encuesta sobre Violencia Doméstica contra las Mujeres de 2014, la tasa de mujeres en Turquía que fueron víctimas de violencia física por parte de sus maridos o parejas en algún momento de sus vidas fue del 36,0%^(12)^. En un estudio realizado en Turquía, se informó de que las noticias sobre violencia aumentaron exponencialmente entre 2013 y 2016^(13)^.

En el presente estudio, se descubrió que un total de 1982 mujeres fueron asesinadas en los periódicos examinados en los últimos cinco años. Si se calcula por años, 361 mujeres fueron asesinadas en 2018, 409 en 2019, 421 en 2020, 404 en 2021 y 387 en 2022 (Tabla 1). A partir de los datos obtenidos en el estudio, se puede argumentar que los feminicidios se cometieron a un ritmo similar al de la literatura entre 2018 y 2022. En 2020, aproximadamente 47 000 mujeres y niñas fueron asesinadas por sus parejas íntimas u otros miembros de su familia en todo el mundo. En otras palabras, en promedio, una mujer o una niña es asesinada por un miembro de su propia familia cada 11 minutos^(14)^. Las estimaciones de la prevalencia a lo largo de la vida de la violencia por parte de la pareja íntima son del 20% en el Pacífico occidental, del 22% en los países de ingresos altos y en Europa, del 25% en las regiones de las Américas de la OMS, del 33% en la región de África de la OMS, del 31% en la región del Mediterráneo oriental de la OMS y del 33% en la región del Sudeste Asiático de la OMS. Más de una cuarta parte de las mujeres de entre 15 y 49 años de edad en todo el mundo han sufrido violencia física y/o sexual por parte de su pareja al menos una vez en su vida^(15)^.

En consonancia con los datos de la bibliografía, las mujeres asesinadas tenían en su mayoría entre 20 y 40 años (52,6%) (Tabla 2). En estudios realizados en Turquía en diferentes momentos, los rangos de edad de los feminicidios se determinaron como 21–50^(16)^, 25–39^(13,17)^, 10–20^(18)^, 18–40^(19)^ y 18–30^(20)^, respectivamente. Según los estudios, se puede afirmar que las mujeres están expuestas a la violencia tanto en su juventud como en la edad adulta.

Estudios anteriores indican que las mujeres más expuestas a la violencia son las casadas^(20)^ o las solteras^(11,17, 18, 19)^. En el presente estudio, el 60,3% de las mujeres asesinadas estaban casadas y el 39,7% eran solteras (Tabla 2). Se puede argumentar que las mujeres fueron víctimas de feminicidio por violencia o asesinadas independientemente de su estado civil.

En el presente estudio, el 60,3% de las mujeres asesinadas procedían de familias nucleares y el 39,7% de familias extensas (Tabla 2). Factores como el hecho de que las niñas y los niños fueran criados según valores patriarcales, la incompatibilidad de los cónyuges, un entorno propicio a la violencia y un bajo nivel socioeconómico pueden haber influido en el aumento de la violencia contra las mujeres.

Estudios anteriores también muestran que las mujeres víctimas de violencia feminicida tenían un empleo que generaba ingresos^(19)^, se desconocía su ocupación^(21)^ o no se informó sobre su ocupación^(20)^. En el presente estudio, se determinó que el 68,7% de las mujeres asesinadas eran amas de casa (Tabla 2).

Diferentes estudios proporcionan importantes hallazgos sobre el nivel educativo de las mujeres víctimas de violencia. En un estudio se encontró que el nivel educativo del 87% de las mujeres víctimas de violencia no se incluía^(19)^, mientras que otro estudio reveló que el 77,8% de las mujeres analfabetas y el 51,1% de las graduadas de primaria eran víctimas de violencia^(22)^. En otro estudio, se informó de que solo el 10,5% de las mujeres víctimas de violencia eran graduadas universitarias^(23)^. Los estudios también indican que el nivel educativo de las mujeres víctimas de feminicidio por violencia no se suele mencionar en las noticias de los periódicos^(20)^. En el presente estudio, se descubrió que una parte significativa de las mujeres víctimas de feminicidio en las noticias (77,9%) eran graduadas de primaria (Tabla 2).

Además, el 34,2% de las mujeres fueron asesinadas por sus maridos, el 20,7% por personas no identificadas, el 10,6% por sus hijos, el 9,6% por sus amantes y el 6,2% por sus exmaridos (Tabla 3). Los estudios también indican que la persona que comete el feminicidio suele ser la pareja de la mujer (marido, exmarido, prometido, amante)^(2,17,24, 25, 26)^. Por consiguiente, se puede concluir que las mujeres son víctimas de feminicidio en su mayoría por parte de personas con las que han establecido una relación emocional cercana.

En el presente estudio, la tasa más alta de mujeres asesinadas fue por armas de fuego, el 19,7% por armas blancas y el 6,2% por estrangulamiento, respectivamente (Tabla 4). Los estudios sobre los incidentes de violencia publicados en los periódicos en diferentes fechas muestran que la fuerza física es el medio más utilizado para ejercer violencia contra las mujeres, seguido de las armas de fuego (17,19,20). En otro estudio basado en informes periodísticos durante el periodo de la pandemia, se descubrió que la mayoría de las mujeres fueron asesinadas con armas de fuego (2). A partir de estos resultados, se puede afirmar que las mujeres fueron asesinadas con armas de fuego, fuerza bruta y lesiones.

En estudios anteriores, las mujeres fueron asesinadas por motivos como el deseo de la mujer de divorciarse o separarse^(25,26)^, los celos, la infidelidad, el honor y la separación, las discusiones^(2)^, la sospecha de infidelidad hacia su marido, la solicitud de divorcio o separación^(24)^, etc.^(13,17,20)^. En el presente estudio, el 45,5% de las mujeres fueron asesinadas por discusiones, el 13,4% por rechazo, el 12,4% por solicitar el divorcio y el 11,5% por celos (Tabla 4). La concepción tradicional aceptada en la sociedad como justificación del feminicidio también es responsable de los suicidios y asesinatos de muchas mujeres^(2,27)^.

Cuando se examinaron las tasas de exposición a la violencia en Turquía por regiones, se determinó que la violencia física es más común en la región de Anatolia Central (43%), la violencia sexual es más común en la región de Anatolia Nororiental (16%) y la violencia emocional es más común en la región de Anatolia Occidental (54%)^(28)^. En el presente estudio, se determinó que las regiones donde se cometieron los asesinatos fueron la región de Mármara (28.7%), la región mediterránea (17,2%), la región de Anatolia Central (14,7%), la región del Egeo (10,6%), la región del Mar Negro (9,7%), la región de Anatolia Sudoriental (9,6%) y la región de Anatolia Oriental (9,5%), respectivamente (Tabla 4). Esto sugiere que la región de Mármara puede estar relacionada con el hecho de ser una región cosmopolita que acoge a migrantes y tiene una alta densidad de población. Sin embargo, se recomienda investigar más a fondo por qué ciertas regiones tienen tasas más altas, teniendo en cuenta factores como la urbanización, la situación socioeconómica y el contexto cultural.

En un estudio realizado en 2021, se descubrió que las mujeres estaban más expuestas a la violencia durante el día^(2)^, mientras que otro estudio reveló que la tasa más alta de violencia se daba por la noche^(20)^. En el presente estudio, se descubrió que la tasa más alta de feminicidios (61.7%) se producía durante el día (Tabla 4).

Es necesario destacar que la violencia contra las mujeres en Turquía es ejercida por hombres. Estudios anteriores muestran que el 29% de los que cometen actos violentos tienen una profesión y el 71% no tienen un trabajo que les proporcione ingresos^(21)^. En un estudio realizado en 2005, se informó de que el 37,8% de los hombres que cometían actos violentos no tenían un trabajo fijo^(29)^. En nuestro estudio, se observó que el 33,2% de los hombres que cometían actos violentos estaban desempleados (Tabla 3).

Los datos del estudio revelan que el feminicidio es un importante problema de salud pública en Turquía y que es necesario adoptar medidas urgentes. La prevención de la violencia contra las mujeres es fundamental no solo para la protección de los derechos individuales, sino también para la protección de la salud y el bienestar general de la sociedad.

Los profesionales de la salud, especialmente las enfermeras, son actores importantes en términos de intervención temprana, orientación y sensibilización, ya que tienen contacto directo con las mujeres víctimas de violencia. Por lo tanto, deben aumentarse los programas de capacitación para el personal de salud. Además, las noticias sobre el feminicidio en los medios de comunicación deben presentarse de manera sensible a la dignidad humana y los derechos de las mujeres, y deben estar respaldadas por regulaciones legales.

### Limitaciones

Los datos del estudio se obtuvieron únicamente de periódicos de Turquía que eran de libre acceso en línea entre 2018 y 2022. Por este motivo, no se pudieron examinar las noticias sobre feminicidios en medios impresos de pago o de acceso limitado, ni en otras fuentes de noticias. La falta o insuficiencia de información sobre algunos acontecimientos en las noticias evaluadas limitó el análisis detallado. Las estadísticas obtenidas se limitan a la información publicada en los periódicos y pueden no coincidir totalmente con los datos reales.

Este estudio se basa únicamente en informes de feminicidios en sitios web de noticias en línea. Por lo tanto, es posible que no reflejen la exhaustividad, la precisión y la cobertura que proporcionan los datos epidemiológicos oficiales. Estos resultados no pueden generalizarse a la sociedad. Las fuentes de datos de otros estudios utilizados en evaluaciones comparativas pueden haberse obtenido de fuentes más sistemáticas, como registros oficiales o informes de medicina forense. Estas diferencias metodológicas pueden limitar la comparación directa de los resultados de nuestro estudio. Es necesario ampliar las limitaciones de las fuentes de datos y reconocer que los medios de comunicación en línea pueden diferir de los medios impresos en términos de información y audiencia.

La herramienta de recopilación de datos utilizada en este estudio no se probó previamente y no se realizó un análisis de validez. Esto puede suponer una limitación en cuanto a la fiabilidad de los datos obtenidos. Sin embargo, las variables se definieron claramente y los datos fueron recolectados por dos investigadores independientes para reducir el margen de error.

## CONCLUSIÓN

En el estudio se identificaron un total de 1982 noticias sobre feminicidios. En el presente estudio, que examinó los feminicidios en las noticias de los periódicos, se descubrió que, en 2020, las mujeres fueron asesinadas principalmente por sus maridos, con armas de fuego, a causa de discusiones, por ser graduadas de primaria, casadas y amas de casa, en familias extensas, en la región de Mármara, entre los 20 y los 40 años, durante el día y por hombres que trabajaban. Los resultados revelan que los feminicidios se basan en causas multidimensionales y que es necesario desarrollar estrategias de prevención integrales.

## Data Availability

El conjunto completo de datos que respalda los resultados de este estudio está disponible previa solicitud al autor correspondiente.
